# Revolutionizing healthcare and medicine: The impact of modern technologies for a healthier future—A comprehensive review

**DOI:** 10.1002/hcs2.115

**Published:** 2024-10-09

**Authors:** Aswin Thacharodi, Prabhakar Singh, Ramu Meenatchi, Z. H. Tawfeeq Ahmed, Rejith R. S. Kumar, Neha V, Sanjana Kavish, Mohsin Maqbool, Saqib Hassan

**Affiliations:** ^1^ Department of Research and Development Dr. Thacharodi's Laboratories Puducherry India; ^2^ Department of Biotechnology, School of Bio and Chemical Engineering Sathyabama Institute of Science and Technology Chennai Tamilnadu India; ^3^ Department of Biotechnology, SRM Institute of Science and Technology Faculty of Science and Humanities, Kattankulathur Chengalpattu Tamilnadu India; ^4^ Sidney Kimmel Cancer Center Jefferson Health Thomas Jefferson University Philadelphia Pennsylvania USA; ^5^ Future Leaders Mentoring Fellow American Society for Microbiology Washington USA

**Keywords:** Artificial Intelligence, healthcare, machine learning, multi‐omics, precision medicine

## Abstract

The increasing integration of new technologies is driving a fundamental revolution in the healthcare sector. Developments in artificial intelligence (AI), machine learning, and big data analytics have completely transformed the diagnosis, treatment, and care of patients. AI‐powered solutions are enhancing the efficiency and accuracy of healthcare delivery by demonstrating exceptional skills in personalized medicine, early disease detection, and predictive analytics. Furthermore, telemedicine and remote patient monitoring systems have overcome geographical constraints, offering easy and accessible healthcare services, particularly in underserved areas. Wearable technology, the Internet of Medical Things, and sensor technologies have empowered individuals to take an active role in tracking and managing their health. These devices facilitate real‐time data collection, enabling preventive and personalized care. Additionally, the development of 3D printing technology has revolutionized the medical field by enabling the production of customized prosthetics, implants, and anatomical models, significantly impacting surgical planning and treatment strategies. Accepting these advancements holds the potential to create a more patient‐centered, efficient healthcare system that emphasizes individualized care, preventive care, and better overall health outcomes. This review's novelty lies in exploring how these technologies are radically transforming the healthcare industry, paving the way for a more personalized and effective healthcare for all. It highlights the capacity of modern technology to revolutionize healthcare delivery by addressing long‐standing challenges and improving health outcomes. Although the approval and use of digital technology and advanced data analysis face scientific and regulatory obstacles, they have the potential for transforming translational research. as these technologies continue to evolve, they are poised to significantly alter the healthcare environment, offering a more sustainable, efficient, and accessible healthcare ecosystem for future generations. Innovation across multiple fronts will shape the future of advanced healthcare technology, revolutionizing the provision of healthcare, enhancing patient outcomes, and equipping both patients and healthcare professionals with the tools to make better decisions and receive personalized treatment. As these technologies continue to develop and become integrated into standard healthcare practices, the future of healthcare will probably be more accessible, effective, and efficient than ever before.

## Graphical abstract

The way medical professionals diagnose, treat, and manage diseases has changed dramatically due to several innovative technologies that have emerged in recent years. Thanks to sophisticated algorithms that examine enormous volumes of patient data to find patterns and predict outcomes, artificial intelligence (AI) and machine learning (ML) have become increasingly potent instruments that enable more precise diagnoses. Telemedicine has increased access to healthcare, particularly in rural regions, by facilitating remote monitoring and consultations. Furthermore, wearable technology and IoMT have enabled people to track their health parameters in real‐time, offering insightful information to patients and healthcare professionals. Moreover, personalized therapies have become possible thanks to genomics and personalized medicine developments, which use a patient's genetic information to create exact medications. Together, these technologies have improved healthcare delivery systems’ efficiency and patient‐centeredness while raising the standard of care.

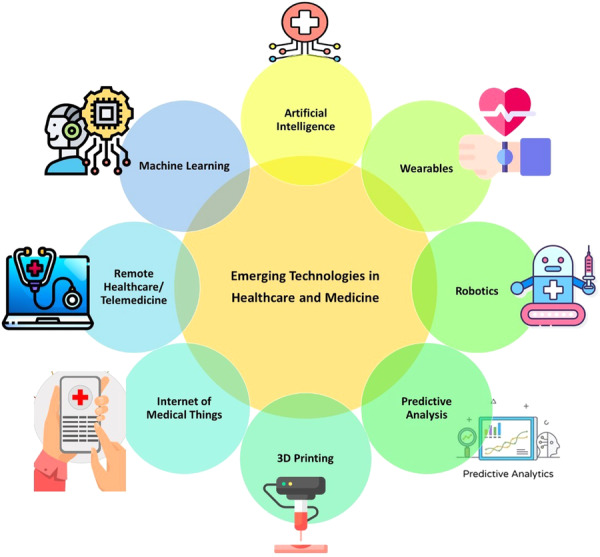

**Abbreviations**
3DP: 3D Printing
AI: Artificial Intelligence
BDA: Big Data Analytics
CAD: Computer‐Aided Design
EHRs: Electronic Health Records
EMRs: Electronic Medical Records
HCPs: Healthcare Professionals
ICT: Information and Communication Technologies
IoMT: Internet of Medical Things
IoT: Internet of Things
IT: Information Technology
LMICs: Low‐ and Middle‐Income Countries
ML: Machine Learning
NLP: Natural Language Processing
OI: Open Innovation
PUFs: Physical Unclonable Functions
RPM: Remote Patient Monitoring
WSNs: Wireless Sensor Networks

## 1 INTRODUCTION

Healthcare services have significantly improved due to advancements in information technology (IT), especially in the area of remote health monitoring [1]. One of the main goals of using physical sensor networks is the focus on disease prevention and the early detection of high‐risk diseases and disabilities [2]. Modern smart devices and high‐tech equipment, such as wearable and smart wireless sensors, have significantly enhanced the ability to quickly monitor and manage patients' conditions through timely access and continuous evaluation of vital signs [3, 4]. The Internet of Things (IoT), AI, and blockchain technologies have rapidly gained traction in many academic and professional domains, most notably in the healthcare sector [5]. These advancements in healthcare delivery have provided many individuals with access to sophisticated, tailored healthcare, thereby improving their quality of life. Given that healthcare is a critical sector that deals with human lives on a daily basis, the implementation of technological interventions has become essential to enhance the efficacy of medical care. Comparative analyses of the recent publications related to the use of modern technologies in healthcare are summarized in Table 1.

**Table 1:** Comparative analysis of the recent publications related to use of the modern technologies in healthcare.

Title of the article	Aim	Findings/Results	Conclusions	References
Multimodal Healthcare AI: Identifying and Designing Clinically Relevant Vision Language Applications for Radiology	To explore the application of vision‐language models (VLMs) in radiology to enhance clinical workflows. VLMs can assist in generating draft reports from images, facilitating augmented report reviews, and highlighting patient imaging histories	Clinicians prefer tool‐based interactions over chatbots for clinical decision support due to time constraints and trust issues, emphasizing AI's role as an information resource rather than a decision agent	Directing new AI towards specific, practical tasks can ensure effective integration into workflows and the development of useful mental models for AI usage. Researchers discovered that AI serves as an effective tool for extracting and processing information, supporting human interpretation	[6]
Securing the Future of IoT Healthcare Systems: A Meta‐Synthesis of Mandatory Security Requirements	To identify the necessary security requirements for the implementation of H‐IoT systems using a meta‐synthesis approach	14 security requirements were determined, focusing on quantitative and technical properties	Addressing these security requirements is crucial to safeguarding H‐IoT systems and ensuring resilience in healthcare information security	[7]
Role of Emerging Technologies In Future Iot‑Driven Healthcare 4.0 Technologies: A Survey, Current Challenges and Future Directions	To gain a comprehensive understanding of various emerging technologies and offer insights for future H‐IoT systems	Block chain, edge computing, and machine learning, can greatly improve performance metrics like accuracy and response time while meeting QoS requirements	Interoperability, scalability, affordability, and privacy	[8]
The Role of Artificial Intelligence in Healthcare: A Structured Literature Review	To examine the current state of AI research in healthcare, with an emphasis on diagnostics, predictive medicine, and health services management	Using bibliometric analysis, the study finds five primary clusters related to AI in healthcare while highlighting the advantages of AI enhances diagnosis precision and decision‐making	Significant challenges exist in ethics, data governance, and health workforce competencies	[9]
Explainable Artificial Intelligence for Predictive Modelling in Healthcare	To explore how explainable AI (XAI) can enhance the trust and applicability in healthcare predictive modelling	Transparency concerns and professional trust are vital; XAI can address these concerns	Successful adoption of AI requires both technology advancement and stakeholders involvement	[10]
Enabling Artificial Intelligence of Things (AIoT) Healthcare Architectures and Listing Security Issues	To assess and disseminate information on AIoT medical innovations in healthcare	IoT applications in healthcare are still in their early stages, with limited utilization causing challenges in various subfields	Interdisciplinary integration and security concerns need to be addressed	[11]
Recent Advancements in Emerging Technologies for Healthcare Management Systems: A Survey	To evaluate the application of Smart Sensor, IoT, AI, Blockchain in healthcare management systems	IoT‐assisted wearable sensor systems, AI, and Blockchain, as well as the critical concerns that must be addressed to enhance the application of these emerging technologies in the healthcare management system	Provides a detailed review of these technologies in healthcare management systems	[12]
Advancements and Future Prospects of Wearable Sensing Technology for Healthcare Applications	To discuss the relevance and future development of wearable devices in the customized healthcare	The difficulty of delivering effective real‐time medical services has been demonstrated to be overcome by wearable sensors and related healthcare systems	Few privacy concerns include sensor reading accuracy, and compatibility with medical ecosystems; it may take time for these wearables to be widely adopted	[13]
The Cost‐Effectiveness of Digital Health Interventions: A Systematic Review of the Literature	To summarize the evidence on the cost‐effectiveness of digital health interventions and assess whether the studies meet established quality criteria	Results regarding the cost‐effectiveness of digital interventions indicated a typically positive impact in terms of costs and health outcomes, supported by an increasing body of research. However, it is still challenging to compare different interventions because of the variability in study techniques	Further research based on a standardized approach is needed to methodically analyze incremental cost‐effectiveness ratios, costs, and health benefits	[14]
Application of Internet of Things and Sensors in Healthcare	To discuss different applications, technologies, and challenges related to the healthcare system	The IoT provides services that allow a patient to instantly communicate health‐related data in real‐time with a particular physician and healthcare system. Nonetheless, there are a number of IoT‐related issues that need to be resolved to enhance the healthcare system	By eliminating the insufficiencies in IoT apps and technologies, a more innovative IoT healthcare system can be accomplished with more facilities to the healthcare system in a cost‐effective manner	[15]
The Impact of Telemedicine and Remote Patient Monitoring on Healthcare Delivery: A Comprehensive Evaluation	To evaluate the impact of new technologies on healthcare delivery, focusing on patient outcomes, economic parameters, and overall satisfaction	Primary outcomes revealed significant improvements in patient health. The healthcare costs were found to decrease. Secondary results demonstrated increased patient satisfaction with communication and increase in convenience of services. Accessibility to healthcare services improved, reducing geographic barriers	The study offers strong evidence of the beneficial effects of remote patient monitoring and telemedicine on the provision of healthcare, highlighting the transformational potential of these technologies	[16]
Robots in Healthcare: A Scoping Review	To establish the types of robots being used in healthcare	Advances in technology have made it possible for robots to perform a wider range of intricate tasks in the healthcare sector. According to this review, there are ten primary roles that robots have fulfilled in various healthcare settings. The two main responsibilities were mobility and rehabilitation and surgery	The COVID‐19 demonstrates the changing demands of healthcare, and robots may be able to help with this adaptation	[17]
The Use of Big Data Analytics in healthcare	To analyze the possibilities of using Big Data Analytics in healthcare	The use of Big Data Analytics bring benefits medical facilities	Results of the study confirm that medical facilities are moving towards data‐based healthcare, together with its benefits	[18]
Artificial Intelligence Ethics in Precision Oncology: Balancing Advancements in Technology With Patient Privacy and Autonomy	To provide an overview of earlier research on the application of AI in precision oncology and the ethical concerns raised by this technology is provided	The application of AI to precision oncology holds the potential to completely transform cancer care, ethical concerns should be taken care of	AI in precision oncology could lead to better cancer outcomes. However, the development and application of AI in precision oncology must be based on ethical considerations pertaining to patient privacy, autonomy, and bias prevention	[19]
3D Bioprinting for Next‐Generation Personalized Medicine	To introduce the principles and techniques of bioprinting, focusing on predominant methods including extrusion printing and digital light processing	Bioprinting has emerged as a promising technology in personalized medicine, with rapid progress and promising preliminary results	3D bioprinting has the potential to revolutionize the design of next‐generation personalized medicine with additional technological advancement and biological validation	[20]

Technology has significantly reshaped the dimensions of healthcare in the twenty‐first century. The current use of information and communication technologies (ICT) has introduced efficient patient portals, electronic health records, telemedicine, and more [20]. Technological advancements have led to the development of state‐of‐the‐art medical devices and have provided simple solutions for clinicians and patients [21]. Notably, portable technologies such as wearable devices have contributed to improved illness detection, treatment, and prevention through their advanced integrated circuits and structural innovations, playing a significant role in expanding the diverse fields of healthcare [12].

The emergence of these technologies in healthcare has helped the personalization of patient care, transforming the conventional hub‐based system into a more personalized healthcare management system [5, 22]. In a rapidly changing culture, digital technology advancements aimed at enhancing human health and well‐being must be continuously assessed for their efficacy and efficiency. The World Health Organization defines eHealth as “the economical and secure application of ICTs in support of health and health‐related domains, such as health‐care services, health surveillance, health literature, and health education, knowledge, and research” [13].

This review delves into the multifaceted impact of cutting‐edge technological advancements on healthcare and medicine, exploring their role in shaping a healthier future. A thorough literature search was conducted using reliable databases, with an emphasis on peer‐reviewed journals, conference proceedings, and authoritative reports to collect relevant information.

## 2 TECHNOLOGICAL ADVANCES IN HEALTHCARE

In recent times, healthcare has undergone substantial changes due to the latest technological developments like IoT, AI, 3D printing, blockchain technologies, and large language models (LLMs) [5]. Notably, these innovations have quickly gained attention in the health sector, giving many patients access to advanced personalized healthcare and patient care monitoring, thereby improving their well‐being [5, 23].

### 2.1 Internet of Medical Things (IoMT)

IoMT represents the integration of IoT with medical equipment, creating a network where every medical device can be connected and monitored through the Internet. This technology allows healthcare practitioners to monitor patient care more efficiently and at a lower cost. A recent example of how IoT is leveraged to address global health concerns is during the COVID‐19 pandemic. The need for healthcare to connect with patients virtually and in their homes grew significantly during this period [14]. IoT‐enabled systems effectively monitor patients, addressing problems in healthcare delivery. For instance, IoT‐enabled ambulances are highly effective because they allow remote medical staff to recommend appropriate care, ensuring that patients receive prompt and efficient treatment [14].

Another application of IoT in healthcare is the Barcode and Label system, a wireless cloud platform that links various therapeutic devices for managing and tracking the health of patients with chronic illnesses. This system enables medical teams and mobile healthcare units to respond quickly utilizing real‐time patient data [24].

IoT technology is also employed in Parkinson House, a joint venture between IBM and Pfizer, where sensors installed throughout the house track patients’ movements and transmit the data wirelessly to the attending physician. This enables real‐time monitoring of medication efficacy and adjustments as needed, thereby improving the doctor‐patient connection. Similarly, real‐time monitoring devices for vital signs, such as temperature, blood pressure, and glucose levels, are essential in managing conditions like diabetes [25]. Data collected from these devices are uploaded to a server and shared with the healthcare service provider for further analysis. The sensors are continuously connected to IPV6, and IoT makes it feasible for the patient and the service provider to share data.

### 2.2 Artificial Intelligence (AI) in diagnostics and treatment

AI involves the use of machine intelligence to simulate human capabilities in planning, acting, and accomplishing tasks across various sectors. Machine learning (ML), a subset of AI, uses data and algorithms to mimic human learning processes [26]. AI and ML are increasingly being used in healthcare, assisting providers in a wide range of patient care systems. By increasing the speed, efficiency, and accuracy of diagnostic processes, AI has the potential to revolutionize medical diagnostics [27]. AI algorithms can analyze medical images, such as X‐rays, MRIs, ultrasounds, CT scans, and DXAs, enabling medical professionals to diagnose illnesses more rapidly and accurately. This reduces the burden on healthcare professionals, allowing them to focus more on patient care and meaningful interactions [28].

AI can analyze diverse patient data types, including medical 2D/3D imaging, bio‐signals (such e.g., ECG, EEG, EMG, and EHR), vital signs (e.g., body temperature, pulse rate, respiration rate, and blood pressure), demographic data, medical history, and laboratory test results. This analysis aids in decision‐making and provides precise predictive outcomes, helping healthcare professionals make better‐informed choices regarding patient care [29].

The ageing population and rising prevalence of cardiovascular diseases emphasize the urgency of using emerging technologies to enhance patient outcomes and reduce the overall burden of cardiovascular diseases. Cardiology is one of the medical specialities where AI has already caused a paradigm shift [30].

AI is beginning to demonstrate its ability to identify diseases at an early stage, reducing the risk of disease progression within the healthcare sector [31]. As AI technology continues to evolve, it is poised to become one of the most important fields for future research and development aimed at enhancing healthcare delivery. Research into AI applications for symptoms and lifestyle‐related diseases, as well as pre‐diagnosis tools, has shown tremendous promise in enabling intelligent systems for self‐care. However, despite these advancements, numerous research challenges remain unsolved, particularly concerning the rapid implementation of AI using cutting‐edge tools like deep learning algorithms and natural language processing (NLP) [32].

### 2.3 Telemedicine and remote patient monitoring

Telemedicine, or distance healthcare, includes services provided through audio and video technology, extending healthcare services to remote areas, especially during pandemics, and improving overall healthcare access. Telemedicine enables healthcare providers to offer services, consultations and patient monitoring without being physically present, thereby increasing accessibility [33, 34, 35]. The sharing of health information over remote connections is an essential aspect of telemedicine and remote monitoring. Modern systems can independently gather and compile data, providing it to patients, caregivers, or healthcare providers either locally or through access by healthcare facilities.

Telemedicine and remote patient monitoring (RPM) have the potential to improve patient outcomes, lower healthcare costs, raise provider and patient satisfaction levels, and remove obstacles to healthcare access. As technology advances and healthcare systems evolve, the integration of telemedicine is expected to significantly shape the future of healthcare delivery [15]. According to a study conducted by Motolese et al. [36], telemedicine can be effectively used even in populations with low skill levels and during emergencies like pandemics, which can have a significant impact on the health of neurological patients directly or indirectly (e.g., escalation of stressors) [37]. Numerous studies conducted over the past decade have shown the utility of RPM in enhancing the prognosis of individuals with long‐term medical conditions [38]. For instance, patients with hypertension who monitored their blood pressure at home and communicated their results to their physician saw a notable improvement in blood pressure management [39].

During the COVID‐19 pandemic, RPM programs significantly improved healthcare delivery. Following the pandemic, several hospitals worldwide developed and deployed RPM platforms, many of which centered on monitoring COVID‐19 patients after their discharge from the hospital [40]. Telemedicine has had a significant impact on various aspects of healthcare, particularly in underdeveloped nations. When properly applied, telemedicine can help these nations provide quality healthcare [41, 42]. For instance, in Georgia, EU‐funded telemedicine initiatives have been implemented in 50 rural clinics, enabling the remote monitoring of child development. As digital technology advances to facilitate telemedicine, family physicians in these pilot clinics are also receiving training to operate effectively within the electronic system. Additionally, telemedicine links rural physicians with specialists and colleagues, aiding in the clarification of diagnoses and the formulation of treatment plans. Rural physicians can schedule virtual consultations with experts or convene a virtual council of specialized service providers in advance [43].

In Sweden, nurses are crucial for the delivery of healthcare to rural communities. Small cottage hospitals, which frequently lack a doctor on call during weekends and nights, manage emergency care. However, telemedicine is being utilized to connect nurses with distant physicians, thereby providing specialized treatment to rural populations. The use of telemedicine in Sweden's rural healthcare settings demonstrates how this technology can address the challenges faced by rural communities and increase healthcare access for the rural population [43].

The focus of modern telemedicine and telemonitoring systems has shifted towards developing systems that are efficient, sustainable, and widely accessible. A new generation of plug‐and‐play sensors has emerged alongside devices that gather and process data. Standardizing these sensors can reduce administrative costs and increase their practical use. Despite these advancements, obstacles remain, such as the need for proper training, data ownership and handling, and the applicability of these technologies to larger populations [44].

### 2.4 3D and 4D printing and its applications in health care

3D printing (3DP) technology has revolutionized the development of organ models, bone and joint implants, and precision instruments at the macro scale, leading to improvements in surgical techniques and healthcare [45]. When combined with AI, 3DP can be used to construct complex geometries in plastic or metal with high precision, resulting in improved prototypes, lower costs, shorter processing times, and personalized treatments. An important feature of 3DP is its contribution to precision medicine through personalized treatments [46].

The medical implant industry has already seen significant success with the use of 3DP. Advances in 3D printer technology have made it possible to print intricate biological structures at the microscopic level, while innovations in material design have expanded the range of materials that can be 3D printed. 3DP allows for the creation of complex objects made from metals and polymers using smaller quantities of material. One of the most popular uses of 3DP in the medical industry is the production of anatomical models. The widespread availability of affordable 3D printers and medical computer‐aided design (CAD) software has enabled an increasing number of hospitals to establish 3D printing labs. By using a 3D‐printed model to prepare for surgery, surgeons can reduce time in the operating theater, leading to fewer complications and better long‐term patient outcomes [47, 48]. There is no doubt that 3DP medical devices hold great promise for ingenuity and innovative solutions to persistent and challenging medical issues [49, 50, 51, 52].

Since its incorporation, 3DP has enabled SJD Barcelona Children's Hospital in Barcelona to handle challenging cases. In 2013, the hospital faced a growing number of intricate and risky surgery. To aid in surgical planning, the medical staff turned to 3DP to create precise, comprehensive anatomical models. This approach greatly enhanced the surgical procedure. For instance, using a patient‐specific 3D‐printed model of the pathology, the medical team at SJD Barcelona Children's Hospital investigated several treatment options for a rare pediatric cancer. Ultimately, they opted for an endoscopic procedure, accessing the tumor through the nostrils to remove its upper portion and via the mouth to remove it entirely. This approach was considerably less invasive, spared the patient's important organs and structures, and improved the patient's prognosis [53].

In 2018, doctors at Ochsner Hospital for Children in New Orleans, USA, used 3D anatomical modeling to assist in planning and effectively executing a life‐saving procedure involving complex surgery to reduce atlantoaxial instability—a dangerous point of weakness between the spine and skull. The surgical procedure was carefully planned using the 3D model, ensuring that all team members were aware of the precise techniques and processes needed based on the anatomy of the patient. The outcome was a surgical procedure that went exactly as intended [54].

The use of 3DP also shows great promise in fabricating functional partial finger prosthetics that restore function to amputees. In one case, a 3D‐printed partial prosthetic finger was fabricated, improving the function of the index finger for a male patient with a partial index finger amputation [55]. As 3DP becomes more accessible, it has the potential to revolutionize medical device fabrication, opening up a wide range of new medical applications and devices. Furthermore, 3DP offers an affordable means for patients in developing or low‐income areas to obtain functional finger prosthetics [55]. In another remarkable case, the world's first 3D‐printed PEEK collarbone was successfully transplanted in 2018 by the medical team at Kunming Medical University Hospital in China, in collaboration with the 3D printer manufacturer IEMAI 3D. A 57‐year‐old man with advanced cancer required the removal of his collarbone to eliminate cancer cells from the affected tissues and organs. To avoid potential interference with chemotherapy, the doctors chose a PEEK prosthesis over the conventional titanium mesh to reconstruct the collarbone. The PEEK promised a quicker recovery and showed no adverse effects [56].

In 2018, researchers at Newcastle University in the United Kingdom successfully 3D‐printed the first human cornea. The researchers created a printable “bio‐ink” by combining collagen, alginate, and viable corneal stem cells. Using a basic 3D bioprinter, the bio‐ink was effectively extruded to form the shape of a human cornea in less than 10 min. Customized 3D‐printed corneas were tailored to each patient's requirements by scanning the patient's eye and using the data to produce a cornea that matched the patient's size and shape. Although further testing is needed before 3D‐printed corneas can be used for transplants, this development could eventually alleviate the global shortage of donor corneas [56].

At Cornell University, Jonathan Butcher and his team used 3D tissue printing technology to construct living heart valves that replicate the anatomical structure of native valves. The team created algorithms that automatically generate a whole 3D model of the heart valve from 3D image files of a native valve, enabling the exact production of an artificial valve. Bioprinting using a dual syringe technique with smooth muscle cells, valve interstitial cells, and alginate/gelatin hydrogel mimics the structure of the valve root and leaflets [56].

In several industries, including automotive, aerospace, consumer goods, and healthcare, 4D printing is emerging as a competitive alternative to traditional manufacturing methods. The “fourth dimension” in 4D printing refers to time [46], which enables the creation of products that can change shape or function over time. 4D printing technology allows for the customization of organ components to match a patient's unique anatomy. This technology can be used to print a variety of medical applications, including earlobes, exoskeletons, eyeglasses, windpipes, stem cells, jawbones, cell cultures, vascular networks, blood vessels, tissues, and organs. Additionally, 4D printing can be employed to develop novel dosage forms and drug delivery systems [46].

### 2.5 Robotics in medicine and health sciences

Robots have the potential to completely transform the medical field. The growing integration of robotics in medicine is driven by advancements in computing power, miniaturization, and AI [57]. Medical robots are increasingly recognized for their applications in surgery, particularly for the precise manipulation of surgical instruments through small incisions, guided by robots, computers, and software [57]. These systems offer an exact and controlled surgical field, viewed in three dimensions through high‐definition magnified vision. Since its Food and Drug Administration approval in 2000, the da Vinci surgical system has been utilized in more than 6 million surgical procedures globally. The main advantages of robot‐assisted surgery for patients include fewer incisions, less blood loss, and quicker recovery, similar to the benefits of laparoscopic surgery. Robotics also holds promise for replacing traditional endoscopy. Small robots can be directed to precise areas to perform tasks such as obtaining a biopsy or cauterizing bleeding vessels. Microrobots could be used to enter blood vessels to deliver medication or radiation therapy to a targeted area. Additionally, robotic endoscopic capsules that can be swallowed may patrol the digestive system, collecting data and transmitting diagnostic information back to the operator.

Robotic nurses are being developed to help busy healthcare providers by performing tasks including digital entry, patient monitoring, blood drawing, and cart pushing. An exciting area of medical robotics is the potential to replace antibiotics by using nanorobots equipped with receptors that bacteria can attach to, allowing for targeted treatment of local infections or bloodstream infections. Given the shortage of human resources, it is critical to concentrate on robots that have the potential for widespread use and significant impact, especially during a crisis [16].

Moreover, surgeons may soon be able to handle robots using haptic gloves or other devices, which will provide tactile feedback to the operating physician from a distance. The low (1 ms) latency promised by 5G technology will make such future medical procedures possible [22].

### 2.6 Use of large language models (LLMs) in medicine

Computational AI methods are used by large language models (LLMs) to produce human‐like language [1, 2]. These models can perform tasks such as storytelling, summarization, translation, and answering questions after being trained on vast amounts of data, including that found on the internet. LLMs, specifically designed to process and produce text, garnered significant attention with the public release of OpenAI's ChatGPT in November 2022. LLMs often possess text summarization, paraphrasing, and translation skills that closely resemble human capabilities. The ability to interact actively with models like ChatGPT has made LLMs appealing instruments across multiple domains, including medicine. However, while these models democratize medical knowledge and facilitate access to healthcare, they also pose risks related to scientific misconduct due to a lack of accountability and transparency.

LLMs are expected to significantly influence clinical practice, medical education, and research. However, it is crucial to recognize and account for their limitations. LLMs have been demonstrated to replicate pre‐existing biases and are prone to disseminating misinformation and exhibiting hallucinations [58]. In medical and nonmedical education settings, students are vulnerable to false information, potentially hindering the development of critical thinking skills. Currently, no systems are in place to guarantee that LLMs produce accurate results, which severely restricts their use of LLMs in clinical settings, where errors and misinformation could have lethal consequences.

The lack of accountability associated with LLMs exacerbates these concerns. Additionally, while safety measures are embedded in LLMs to mitigate bias, these measures may fail to recognize certain symptoms in men and women, potentially leading to disparities in treatment. Nonetheless, newer iterations and models created specifically for medical use and trained on medical data demonstrate promising advancements in addressing these issues [59, 60, 61]. However, several prerequisites must be met before LLMs can be widely adopted in the medical field, including addressing concerns about safety, validity, and ethics.

## 3 ADVANCEMENTS IN METHODOLOGIES AND DATA PROCESSING FOR IMPROVED HEALTHCARE

The application of big data analytics (BDA) holds tremendous potential for healthcare, with worldwide significance. BDA has enabled the application of new technology for patient treatment and health management [17].

### 3.1 Multi‐omics

The term “multi‐omics” refers to a research approach that combines several “omics” data sets from various fields of study, including proteomics, metabolomics, transcriptomics, genomics, and epigenomics [62]. With advancements in high‐throughput technology and data science, it is increasingly feasible to leverage these diverse data types simultaneously. Each of these data types offers unique insights into various aspects of a biological system. The novel integration of different omics data, known as multi‐omics, has been facilitated by advances in computing capabilities and omics technologies, such as proteomics and metabolomics. This integration allows for a comprehensive understanding of the complex molecular interactions underlying health and disease by combining the strengths of individual data types [63]. Table 2 lists various multi‐omics databases used for research in precision medicine

**Table 2:** Multi‐omics databases for research in precision medicine.

Consortium	Latest release Status	Omics assays	References
**Type: Cancer**			
COSMIC v99	28‐Nov‐23	Genomics, Transcriptomics, Epigenomics	https://cancer.sanger.ac.uk/cosmic
Genomic Data Commons Data Portal 39.0	04‐Nov‐23	Genomics, Transcriptomics, Epigenomics	https://portal.gdc.cancer.gov/
NCI Proteomic Data Commons V1.6.8	16‐Nov‐23	RNA‐seq, miRNAs, global proteome	https://pdc.cancer.gov/pdc/
Therapeutically Applicable Research to Generate Effective Treatments (TARGET)	2016	Genomic, transcriptomic, and Epigenomic data	https://www.cancer.gov/ccg/research/genome-sequencing/target/using-target-data
TIMER2.0	2020	Epigenomics Transcriptomics Proteomics Immunomics	http://timer.cistrome.org/
Gene Expression Profiling Interactive Analysis (GEPIA)	2021	Genomics Epigenomics Transcriptomics Proteomics Immunomics	http://gepia.cancer-pku.cn/
Molecular Taxonomy of Breast Cancer International Consortium (METABRIC)	−	Genomics Transcriptomics Immunomics	https://www.mercuriolab.umassmed.edu/metabric
Human Cancer Models Initiative (HCMI)	−	Genomic, transcriptomic, and Epigenomic data	https://portal.gdc.cancer.gov/projects/HCMI-CMDC
**Type: Alzheimer's Disease**			
The Alzheimer's Disease Neuroimaging Initiative (ADNI)	2004	Genetic, magnetic resonance imaging, and positron emission tomography imaging	https://adni.loni.usc.edu/
**Type: Parkinson's Disease**			
Parkinson's Disease Biomarker Discovery (PDBD)	2018	Transcriptomics, epigenomics, whole genome sequencing, metabolomics, and proteomics	https://pdbp.ninds.nih.gov/
Parkinson's Progression Markers Initiative (PPMI)	2018	Transcriptomics, epigenomics, whole genome sequencing, metabolomics, and proteomics	https://www.ppmi-info.org/
**Type: Neuropsychiatric disease**			
PsychENCODE	2015	Whole genome sequences, Transcriptomics	https://psychencode.synapse.org/
NIMH Repository and Genomic resources	−	RNA and DNA sequencing, genotyping, epigenetics	https://www.nimhgenetics.org/
**Type: Human genetic variations**			
The International Genome Sample Resource (IGSR)	2007	Whole genome sequences, Targeted Exome sequencing	https://www.internationalgenome.org/
UK Biobank	2007	Genotyping, Whole genome sequences	https://www.ukbiobank.ac.uk/
Functional Annotation of the Mammalian Genome (FANTOM)	2000	RNA‐seq, RADICL‐seq, Cap Analysis of Gene Expression	https://fantom.gsc.riken.jp/
The Genotype‐Tissue Expression (GTEx)	2010	Whole genome sequences, WES, and RNA‐Seq	https://www.gtexportal.org/home/

Multi‐omics integrative techniques have enabled deep phenotyping of individuals in health and disease, leading to numerous clinically valuable findings [64, 65, 66]. Advances in exploring human genomes, proteomics, lipidomics, and metabolomics are driving multi‐omics‐enabled precision health [67].

Despite the challenges in integrating and translating multi‐omics data into relevant functional insights, there is a strong trend toward incorporating multi‐omics analysis into healthcare research to explain the complex relationships across molecular levels. Multi‐omics data can be used to track medical histories, identify patterns, predict outcomes, and enhance prevention, early detection, and personalized treatment planning [68].

### 3.2 Utilization of big data for predictive analytics

BDA involves methods and tools for extracting insight from large amounts of data. BDA outcomes can be applied to predict future events and identify historical patterns. In healthcare, BDA has facilitated the examination of enormous datasets, including information from hundreds of patients, identifying patterns and correlations within the data, and creating predictive models through data mining techniques [69]. BDA is anticipated to lower operating expenses and enhance the quality of life in the healthcare sector [70, 71]. The global paradigm for managing healthcare has shifted from a disease‐centered approach to a patient‐centered approach, even within value‐based healthcare delivery models [72]. To meet the requirements and deliver efficient patient‐centered care, healthcare big data must be managed and analyzed effectively.

### 3.3 Enhancing patient outcomes through data‐driven approaches

Recent studies have emphasized the necessity and significance of creating data‐driven strategies to enhance patient care and safety [73, 74]. Data‐driven technologies operate by gathering, utilizing, and analyzing patient data through the application of AI and ML. These technologies support better patient and public healthcare delivery by utilizing and expanding the depth and breadth of electronic health data [75]. Among the appropriate data sources for data‐driven technologies are electronic medical records. In the rapidly evolving healthcare sector, using data‐driven strategies is becoming increasingly common and essential. These strategies are revolutionizing healthcare delivery, greatly enhancing operational efficiency and patient outcomes. This paradigm shift toward a more analytical approach in healthcare improves the accuracy of medical interventions and paves the way for more personalized patient care.

## 4 ACCESSIBILITY AND EQUITY IN HEALTHCARE

“Health equity” refers to the principle that everyone should have an equal opportunity to achieve optimal health and access the best possible healthcare, irrespective of their financial situation, social standing, geographic location, or other factors. It serves as a key indicator of the effectiveness of a health system and the progress of social development. Numerous nations have made significant efforts to reduce health inequalities across various demographic groups [76, 77, 78, 79]. Access to healthcare is a multifaceted concept involving the procedures that govern an individual's or a population group's entry into the healthcare delivery system. Key dimensions of healthcare access include availability, acceptability, affordability, geographic accessibility, and accommodation [80]. Despite progress, health disparities remain prevalent even in developed nations, while low‐ and middle‐income countries (LMICs) face more challenging obstacles that require sustained efforts [81, 82, 83, 84, 85]. The World Health Organization continues to stress the importance of “leaving no one behind,” underscoring the ongoing need for efforts to achieve health equity despite notable advancements in this area over the past decade [86, 87].

**Figure 1:**
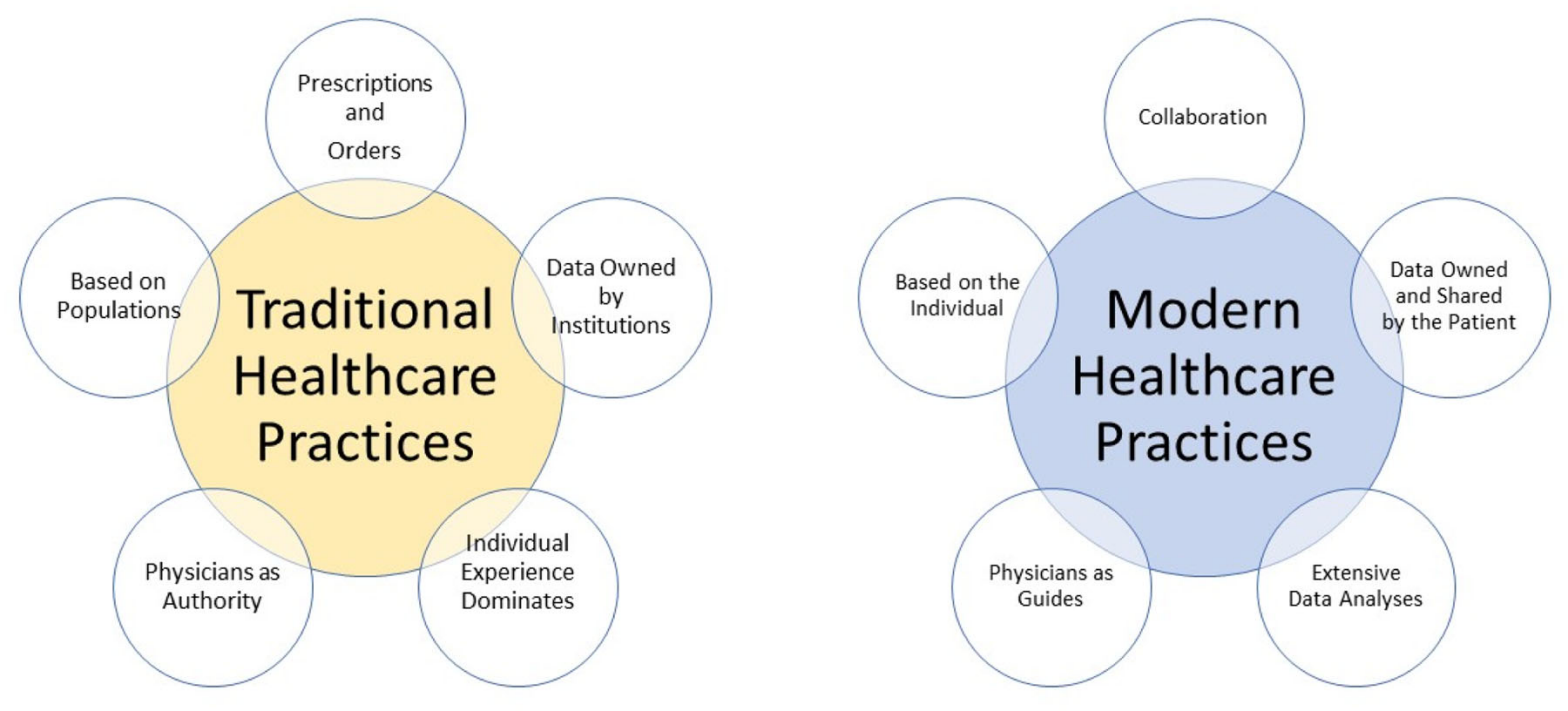
Comparison between traditional healthcare practices and modern healthcare practices (digital health and technological revolution in healthcare).

The rapid advancement of technology plays a crucial role in improving accessibility and equity in healthcare. Traditionally, patients had little involvement in decisions about their health and disease management, with healthcare professionals bearing the full responsibility for medical decisions and outcomes. Patients relied on the procedures, resources, data, and decisions of healthcare institutions and professionals (Figure 1). The growing availability of new technologies, coupled with a sense of vulnerability and exposure to decisions made without their consent, has driven patient empowerment [88]. Digital interventions have indicated a generally positive impact in terms of cost‐effectiveness and health outcomes. For example, s study by the University of Michigan found that switching from paper to electronic health records resulted in a 3% decrease in outpatient care costs, equating to a monthly savings of $5.14 per patient—a significant amount in large urban hospital networks [89]. However, comparing different interventions remains challenging due to variability in study methodologies. More studies using standardized methodologies are needed to thoroughly examine costs, health benefits, and incremental cost‐effectiveness ratios [90]. Addressing healthcare disparities through technology and improving access to healthcare in underserved areas are key areas where these interventions can make a meaningful impact.

### 4.1 Addressing healthcare disparities through technology

Health information technology (IT) can play a significant role in reducing inequities within clinical care settings. The June 2019 supplement of *Medical Care*, titled “Addressing Health Disparities Through the Utilization of Health IT,” discusses how health IT can be used to improve patient‐clinician communication and increase access to treatment, thereby lowering inequalities. The research highlights the significance of utilizing health IT tools, such as mobile technologies, e‐prescribing, electronic health records, and personal health records, to reduce gaps in healthcare outcomes and access [91]. Additionally, health IT can improve patient‐clinician interaction by providing language and literacy‐appropriate information and visual aids, benefiting those with limited English proficiency and low health literacy [92].

### 4.2 Improving access to healthcare in underserved areas

According to a study by the Association of American Medical Colleges, the lack of doctors is expected to range between 37,800 and 124,000 over the next decade, with a projected shortfall of around 50,000 primary care physicians during this period [93]. Significant advancements in technology are now available to support doctors in providing care to patients outside their offices. Telehealth and telemedicine are being expanded to improve access to primary care in underserved and rural populations. These technologies can increase access to treatment, improve health outcomes, lower inequities, and offer virtual consultations. Millions of individuals living in rural areas can benefit from virtual follow‐up visits, medication monitoring, and a variety of therapeutic services, allowing them to receive care in the comfort of their own homes [94].

A study by Doximity involving 2400 adult patients and 1200 physicians examined the motivations behind the use of telemedicine and its effects on access to and quality of healthcare. According to Jeff Bendix's article “The State of Telemedicine Today,” 40% of Americans use telemedicine daily and 44% use it weekly. The majority (95%) of these interactions include video, and 88% of respondents believe telemedicine has considerably enhanced access to healthcare, largely due to its ease of use (92%) [95]. In addition, digital health technologies have the potential to improve patient empowerment, improve compliance and adherence, and overcome geographic barriers. These benefits could include easier access to healthcare services, more direct communication with healthcare providers, and full access to information storage and sharing for better follow‐up and clinical decision‐making [96, 97].

### 4.3 Accessibility and affordability of digital health technology in low‐resource settings

Post‐COVID‐19, digital technologies and health data have become crucial for enhancing health outcomes worldwide. LMICs, in particular, hold promise, given their emerging digital health policies and the numerous obstacles they face. LMICs must collaborate with international partners on fundamental elements of a global digital health framework, such as national plans, skills, ICT infrastructure, and governance that balances innovation and data security. Nations ought to create comprehensive national plans for digital health. The state of development varies greatly among LMICs, and several significant nations lack official national plans. Without a plan that offers the necessary resources, organization, cooperation, and leadership, digital technologies will fall short of realizing their full potential. These strategies must be comprehensive as the circumstances in each nation will vary, especially when it comes to the complex task of integrating digital technologies with established health systems.

### 4.4 Digital healthcare technologies‐regulatory landscape and policy considerations in low and middle‐income countries

Governments should develop thorough national plans for digital health. LMICs differ widely in their level of development, and several important countries lack formal national plans. Without strategic plans that provide the necessary funding, coordination, leadership, and cooperation, digital technologies will never reach their full potential. The complexity of integrating digital technology with established health systems requires that these policies be comprehensive and tailored to each country's unique conditions. Although digital literacy training and education are essential, few LMICs have included digital skills into their health workforce training programs. Donors, regional and multilateral health organizations, and other stakeholders should prioritize initiatives to help LMICs address critical skills shortages [98].

The majority of the world's disconnected population resides in LMICs, where ICT infrastructure deficiencies are particularly severe. The potential of digital health is greatly limited by inadequate ICT infrastructure. To address these gaps, donors, regional and multilateral development organizations should intervene where the private sector falls short, such as in rural wireless mobile coverage.

LMICs must implement a data governance framework that strikes a balance between innovation and data privacy and protection. The creation, safeguarding, application, dissemination, and global exchange of high‐quality data are essential to a successful and innovative digital health initiative. However, a data governance system that is too rigid could impede the potential of digital health technology [98].

Interoperability must be incorporated into policy frameworks from the outset, as many of the advantages of digital health technology depend on cross‐border data flows. This is crucial because many companies and research groups in digital health rely on the Internet, open data exchange, and centralized IT infrastructure to access patient information, data, and healthcare providers globally in an accessible, affordable, and reliable manner. The establishment of a comprehensive and cohesive worldwide digital health structure will depend on national governments permitting transnational data transfers [98].

## 5 BALANCING TECHNOLOGICAL ADVANCEMENT WITH ETHICAL CONSIDERATIONS

### 5.1 Precision medicine and privacy concerns

Precision health offers numerous benefits, but it also raises privacy risks due to the sharing of personal data across potentially unreliable channels. Digital technologies and advanced data analysis have the potential to revolutionize translational research, but their approval and adoption pose scientific and regulatory challenges [99, 100]. New technologies, such as liquid biopsy, are proving beneficial in disease diagnosis and treatment, especially in cancer care, where precision medicine leverages advancements in molecular technologies. While AI can reduce repetitive tasks and improve patient‐physician relationships, it must be balanced with ethical considerations, focusing on patient privacy and avoiding bias [18, 101].

Large studies have identified genetic associations in diseases, and stratified medicine groups patients based on biomarkers to improve treatment outcomes [102]. Precision medicine aims to tailor treatments to individuals based on molecular features [103]. Advances in functional genomics now allow for the measurement of more proteins and metabolites, driving the growth of personalized medicine.

Genotype‐guided treatment is one of the most extensively researched applications of precision medicine in healthcare today, helping clinicians determine the appropriate dosage based on genotype information [104]. For patients with lung or breast cancer, genomic profiling of malignancies can inform tailored treatment regimens [105]. Incorporating precision medicine into healthcare can lead to more accurate diagnoses, identify patients at risk before symptoms appear, and provide individualized treatment plans that balance effectiveness and safety.

Wearables and mobile technologies have seen significant adoption and innovation, tracking various parameters and promoting active participation in health and wellness [106, 107, 108]. The big data generated by wearables and multi‐omics profiling offers potential for behavioral change, representing a significant shift towards personalized nutrition and precision medicine. Electronic medical records (EMRs) are important for personalized treatment plans, and recent federal provisions have made EMR data more accessible for research. Biomedical imaging informatics supports personalized diagnosis, treatment guidance, biomarker identification, and monitoring through imaging data analysis. AI technology in surgery holds the potential to provide more precise, efficient, and personalized interventions [109].

### 5.2 Opportunities for innovation and collaboration in healthcare

The healthcare sector is increasingly adopting open innovation (OI) to drive innovation in contemporary settings, but several obstacles remain, including skepticism among experts, intellectual property rights concerns, and established procedures. Implementing OI requires new ways of thinking, which challenge organizational culture and structure. The healthcare industry is distinct because it deals with biological knowledge and is mastered by experts, and this complex and fragmented environment hinders innovation and information transfer [110].

Through innovation, the healthcare sector is moving towards proactive prevention, and collaboration is essential for this transformation. OI projects can improve resource utilization. However, deeper sector‐specific collaboration and experimentation via distributed methods are still necessary. The future of personalized medicine relies on the standardization, integration, and harmonization of technologies. Advancements in ‘omics’ techniques will enable more individualized treatments. Evidence‐based medicine (EBM) uses the best evidence to make decisions about individual patient care. While randomized controlled trials are the “gold standard,” personalized medicine creates customized interventions based on each patient's unique disease characteristics, revolutionizing treatment and focusing on preventive healthcare. Transitioning to individualized medicine requires integrating data to understand organism functioning [111, 112].

Despite the tremendous progress and applications of AI in improving the treatment process, these advancements are not accessible to all societies. Many low‐income and developing countries still lack access to the latest technologies. It is important to emphasize that there are several obstacles to overcome when integrating AI into healthcare, including ethical constraints, privacy and data protection, informed consent, medical consultation, and empathy. Healthcare experts should take into account the four fundamental medical ethical principles – autonomy, beneficence, nonmaleficence, and justice – in all aspects of healthcare before incorporating AI into the system [113, 114, 115].

### 5.3 Challenges and limitations associated with digital health technology and some possible solutions

Innovations in digital health hold great potential to transform healthcare delivery, but developing nations must overcome significant legislative and technological obstacles to fully capitalize on these advancements. Barriers preventing digital health from reaching its full potential include inadequate data security measures, interoperability problems, legislative difficulties, and infrastructure limitations. Addressing these issues is not only advantageous but essential for building resilient, inclusive healthcare systems [116]. The lack of evidence‐based digital health standards, privacy concerns, data governance issues, and ethical challenges are major obstacles impeding the progress of digital health [117, 118, 119]. The use of EHRs and other digital health technologies generates large amounts of data that can be used to create valuable evidence. However, much of this data is collected through convenience sampling [120, 121], leading to potential bias in health data [122]. These biases can impact the effectiveness and impartiality of AI systems [123]. Healthcare providers must guarantee that the data used by AI systems is accurate, secure, and representative of the population in question [124]. This issue influences the overall quality of the data generated by studies using these technologies. To address this problem, it is necessary to report and compare background data between groups, such as age, sex, socioeconomic status, and geographic distribution [125, 126]. The digitalization of health has also led to privacy issues. Digital health platforms produce data that must be protected at every stage. Despite advancements in anonymization technologies, re‐identification remains necessary to correctly combine updated data with the original data belonging to the same individual. The risk of re‐identification hacking makes securing digital health platforms critically significant [125].

Governments also face difficulties in data governance. While many governments are moving toward digitalization due to technology advancements that reduce costs, only about half have privacy laws in place to safeguard citizen data. Therefore, government regulations and standards for data governance are crucial [127]. The digitization of health brings serious ethical challenges, one of which is obtaining informed consent from users. Users ought to be aware that data is being collected, but many applications require consent only through a simple “I agree” button, which users often click without fully understanding the terms of use [128]. Policymakers need to address the sustainability and cost‐effectiveness of digital care. Gaining public trust and demonstrating a commitment to protecting privacy are essential.

The COVID‐19 pandemic accelerated the adoption of technologies like IoMT, allowing doctors to remotely diagnose patients, operate hospital equipment, and monitor patients. However, the sharing of sensitive data over insecure wireless mediums via IoT nodes raises significant security concerns. Secure sessions are necessary to safeguard virtual medical facilities from hostile attacks [129]. Masud et al. [129] proposed a lightweight, physically secure mutual authentication and secret key establishment protocol that allows the network devices to authenticate the doctor (user) and sensor node before generating a session key. This protocol uses physical unclonable functions to protect against side‐channel, cloning, and manipulation attacks on sensor nodes placed in hostile and unsupervised environments. The protocol demonstrates every security feature—such as anonymity, integrity, secrecy, and authentication—needed to secure IoMT networks.

The healthcare industry has seen increased interest in online medical data exchange facilitated by Wireless Sensor Networks (WSNs). However, the implementation of wearable sensor nodes in resource‐demanding applications is restricted by their low energy, storage capacity, and data processing capability. Cloud storage services can improve the capabilities of wearable sensors and offer a useful way for group members to share data. However, as medical data directly connects to patients' health and personal information, protecting the integrity and privacy of medical records stored on cloud servers is a critical issue that needs urgent attention. Xu et al. [130] developed a secure and efficient certificate less public auditing system for cloud‐assisted medical WSNs that allows for effective group user revocation, privacy protection, and dynamic data sharing. Performance evaluations and security analyses show that their system achieves a higher level of security at a lower total computation cost.

AI systems process inputs and produce outputs in a manner similar to human brains, but healthcare professionals (HCPs) typically only see the output without understanding the measurements or reasoning that led to a conclusion – this is known as the “black‐box problem” [131]. Holding medical professionals accountable for AI mistakes could hinder AI adoption. Therefore, guidelines and safeguards should be developed to protect both medical professionals and AI systems. Enhancing healthcare workers' understanding of AI‐related risk and setting realistic expectations for AI performance is emphasized. A user‐friendly AI interface is essential, and the data presented must be relevant to clinical practice. Before developing and promoting AI in healthcare, it is necessary to identify clinical demands and involve all stakeholders, including providers, payers, and patients [131].

## 6 SUSTAINABILITY OF HEALTHCARE TECHNOLOGIES

Decarbonizing the healthcare sector is both a difficult and urgent task, but leveraging technology offers a path toward more environmentally sustainable practices. By integrating technology into the healthcare supply chain with an emphasis on enabling home therapies and creating products and technologies with sustainability in mind, the industry can drastically lower its carbon emissions. Achieving sustainability in healthcare will also require minimizing the environmental impact of products by designing them for easy disassembly and recycling as they reach the end of their useful lives. While single‐use goods will always be necessary due to the hazardous nature of healthcare waste and the requirement for infection control, the industry must strive to reduce, reuse, or recycle components that do not come into direct contact with patients.

In addition to maximizing the benefits of technological advancements in healthcare, increasing the use of wearables, software, digital tools, and virtual aid can save resources. These technologies empower individuals to take a user‐friendly, preventative approach to regularly monitor their well‐being. For instance, wearables like the Viscero ECG Vest allow patients to receive high‐quality care from the comfort of their homes, eliminating the need for visits to resource‐intensive healthcare facilities for diagnosis and treatment. Implementing technologies that lower the carbon footprint of both healthcare providers and patients can enhance patient experiences, combat climate change, and improve overall healthcare sustainability. However, achieving Net Zero and promoting the shift to more environmentally friendly healthcare will require innovations in supply chain management and wearable technologies, developed with a deep awareness of both patient and healthcare professional needs to ensure seamless integration into daily lives [132].

## 7 FUTURE TRENDS AND PREDICTIONS

The future of healthcare is individualized and driven by advancements in nanotechnology, IT, and genetics. Advances in anesthesia and minimally invasive surgical practices are expected to decrease inpatient volumes while increasing outpatient procedures [133]. Advances in oncogenomics and cancer pharmacogenomics, driven by next‐generation sequencing technology, are anticipated to personalize cancer screening and diagnosis [134]. AI systems will shift healthcare from traditional models to cost‐effective, data‐driven disease management strategies, furthering immunomics and drug discovery for better preventive strategies [135]. Wearable healthcare and IoT devices equipped with biosensors and transducers promise continuous health monitoring and reduced hospital visits. Examples include real‐time insulin level monitoring, contact lens‐based tear monitoring, and colorimetric wearables that assess sweat as an alternative biomarker for measuring blood sugar and glucose levels. These wearable devices have the potential to deliver telemedicine and telehealth services while also acting as healthcare data storage devices [12].

Quantum machine learning (QML) trained with clinical datasets, can be used for diagnosing medical imagery, generating novel drug candidates, and predicting treatment efficacy [136]. 3D bioprinting is expected to advance personalized medicine by driving drug development and in‐vitro tissue and organ engineering [19]. AI will assist pathologists in performing repetitive tasks accurately, freeing up cognitive space for cancer prognosis and therapeutic efficacy [137]. The future of diagnostic imaging in radiology includes the integration of machine learning for the automation of magnetic resonance imaging (MRI) and computed tomography (CT) scans, promising enhanced diagnostic capabilities [138]. AI‐driven machine learning can enhance endoscopic diagnosis by distinguishing between cancer and adenoma, detecting colorectal cancer, and utilizing wireless capsule endoscopy. However, quality clinical trials are essential to implement these practices effectively. Virtual GI clinics, home‐based diagnostic testing, and virtual reality therapies are some of the far‐reaching applications of AI integration. The real‐world performance of these technologies may fall short of expectations due to various challenges. Key developments, such as recognizing AI as a medical device, addressing the need for randomized trials, solving the “black box” problem, and establishing regulatory institutions to be held accountable for errors, are necessary to realize this future [139, 140]. As global populations age, many healthcare systems are struggling to cope. Fortunately, technological advancements like AI and telehealth offer hope for tackling some of the most critical issues facing the healthcare industry.

## 8 CONCLUSION

The surge of innovations, encompassing AI, telemedicine, precision medicine, and BDAs, has revolutionized the healthcare sector. The transformative potential of these advancements becomes evident as we gaze into the future of healthcare. The healthcare sector holds tremendous promise, driven by the convergence of state‐of‐the‐art technologies, data‐driven methodologies, and a dedicated commitment to mitigating healthcare disparities. This synergy between healthcare and technology presents exciting possibilities, paving the way for personalized treatments that account for individual variations and ultimately lead to enhanced patient outcomes. Moreover, this technological evolution has the potential to establish a more accessible healthcare system, dismantling barriers and delivering quality medical services to diverse populations, including those residing in underserved areas.

As we move into the next decade, collaborative endeavors between healthcare and technology are poised to redefine our approach to wellness. The journey towards a promising future requires active engagement, innovation, and careful navigation of ethical considerations within the healthcare sector. By embracing these principles, stakeholders in the healthcare ecosystem can collectively contribute to a landscape that is not only technologically advanced and efficient but also profoundly centered on the well‐being of individuals and communities. This transformative trajectory holds the potential to create a healthcare environment that is responsive to the evolving needs of society and dedicated to fostering optimal health outcomes for all.

## AUTHOR CONTRIBUTIONS

**Aswin Thacharodi, Prabhakar Singh, and Ramu Meenatchi**: Writing—original draft; data curation; writing—review and editing; visualization. **Z. H. Tawfeeq Ahmed**: Writing—original draft. **Rejith R. S. Kumar**: Writing—original draft. **Neha V**: Writing—original draft. **Sanjana Kavish**: Writing—original draft. **Mohsin Maqbool**: Writing—original draft; data curation. **Saqib Hassan**: Conceptualization; data curation; supervision, validation; writing—original draft; writing—review and editing.

## CONFLICT OF INTEREST STATEMENT

The authors declare no conflict of interest.

## ETHICS STATEMENT

Not applicable.

## INFORMED CONSENT

Not applicable.

## Data Availability

Data sharing not applicable to this article as no datasets were generated or analyzed during the current study.
